# Challenges for early diagnosis of neonatal herpes infection in Japan

**DOI:** 10.3389/frph.2024.1393509

**Published:** 2024-08-08

**Authors:** Junya Kojima, Shunji Suzuki, Shin-Ichi Hoshi, Akihiko Sekizawa, Yoko Sagara, Hideo Matsuda, Isamu Ishiwata, Tadaichi Kitamura

**Affiliations:** ^1^ Division of Maternal and Child Health, Japan Association of Obstetricians and Gynecologists, Tokyo, Japan; ^2^ Japanese Foundation for Sexual Health Medicine, Tokyo, Japan

**Keywords:** herpes simplex virus infection, neonate: risk factor, Japan, maternal symptom, incidence

## Abstract

**Background:**

This study aimed to analyze the recent prevalence of neonatal herpes simplex virus infection, maternal symptoms in the presence of neonate who has herpes simplex virus infection, and mode of delivery in Japan.

**Methods:**

We requested 2.078 obstetrical facilities that are members of the Japan Association of Obstetricians and Gynecologists (JAOG) to provide information on neonatal herpes simplex virus infection involving deliveries at or after 22 weeks of gestation between 2020 and 2022. Of these, 1.371 (66.0%) facilities responded with information that could undergo statistical analysis.

**Results:**

There were 10 cases of neonatal herpes simplex virus infection, and the incidence of neonatal herpes simplex virus infection in Japan was about 1 in 1.4 × 10^5^ live births. There were no characteristic maternal findings common to cases of neonatal herpes simplex virus infection.

**Conclusion:**

The incidence of neonatal herpes simplex virus infection in Japan was low. We could not identify any characteristic maternal findings common to cases of neonatal herpes simplex virus infection.

## 1 Introduction

Common sexually transmitted infections, such as genital herpes simplex virus (HSV) infection, are spread by close personal contact (1–4). For example, HSV-1 can be transmitted to another person through kissing or oral sex, while HSV-2 can be acquired via vaginal, anal, or oral contact with an infected individual (1–4). When a woman of reproductive age contracts an infection, HSV infection may be vertically transmitted to neonates.

Neonatal HSV infection may be uncommon; however, the morbidity and mortality associated with infection are high (1–4). The global estimate of the incidence of neonatal HSV has been reported to be 10 cases/100,000 live births; however; however, the incidence and mortality rates have been believed to be underestimated (5–7). It develops after an incubation period of 2 to 10 days, and it is characterized by unclear initial symptoms (3). The first symptoms of neonatal HSV infection can vary from fever, small blisters, lethargy, dyspnea, large abdomen due to ascites or large liver, or no symptoms. HSV encephalitis or infections of other organs such as the liver, lungs, and kidneys can result from HSV infection with or without skin symptoms. Neonatal HSV infection typically causes viral pneumonia and intravascular coagulopathy. Neonates who survive severe infection typically can have lifelong neurological conditions. Although most neonatal HSV infections are acquired perinatally through the birth canal, many newborns with HSV infection have been reported to be born to mothers with no prior history of HSV infection or symptoms (1, 3, 8).

Therefore, to prevent trans-vaginal infections if genital lesions are present at delivery, cesarean section has been recommended for pregnant women with HSV infection (1, 2, 8, 9). For example, in the guidelines for obstetrical practice in Japan 2020 (= Japanese guidelines) (9) elective cesarean delivery has been recommended before labor or premature rupture of the membranes under the following conditions: (a) genital lesions of HSV infection recognized or strongly suspected at admission for delivery, (b) delivery within a month after primary HSV infection, and (c) delivery within a week after recurrent or non-primary HSV infection.

In our earlier survey in Japan in 2017, the proportion of women with symptoms of genital HSV infection during pregnancy was 1 in 536, and cesarean delivery indicated for genital HSV infection was performed in 15% (about 280) of the women (10). However, approximately 30% of mothers with neonatal HSV infections have been reported to show symptoms of the disease (1, 3). In addition, in our earlier observation in Japan, none of 6 cases with cerebral palsy due to neonatal HSV infection showed any findings suggestive of HSV infection in mothers (11). Furthermore, it has been suggested to be uncommon for HSV to be transmitted vertically during the preclinical phase of the disease (12, 13). Therefore, it may be important to investigate mothers whose neonates develop neonatal HSV infection.

To evaluate the effectiveness of cesarean section as recommended by the guidelines (9) and re-examine strategies to prevent the development of neonatal HSV infection in the future, this study aimed to analyze the recent status of neonatal HSV infection, maternal symptoms in the presence of a neonate who has HSV infection, and mode of delivery in Japan.

## 2 Methods

### 2.1 Study population

The protocol for this study was approved by the Ethics Committee of the Japan Association of Obstetricians and Gynecologists (JAOG). In March 2023, we requested 2.078 obstetrical facilities that are members of JAOG to provide information on neonatal HSV infection in deliveries at or after 22 weeks of gestation between January 2020 and December 2022. In Japan, all infants are required to undergo a physical examination at the delivery institutes one month after birth, and so obstetricians at the institutes can always be aware of cases with onset of neonatal HSV infection within 10 days after birth. Furthermore, institutes affiliated with JAOG report the number of deliveries each year in detail to the JAOG office, so the incidence of neonatal HSV infection can be calculated based on those figures.

Of the 2.078 facilities, 1.371 (66.0%) facilities responded with information that could undergo statistical analysis. At the 1.371 facilities, 1.433.001 neonates were born during the study period, which corresponded to about 59.2% of the total number of births in Japan (average number of births during the year: about 810.000).

### 2.2 Clinical assessment

The information requested was as follows: maternal age, past history of HSV infection, gestational age at delivery, premature rupture of the membranes, maternal symptoms indicating viral infection around the time of delivery, delivery mode, timing of the onset of neonatal HSV infection, disease type and HSV type of neonatal HSV infection.

## 3 Results

### 3.1 Incidence of neonatal HSV infection in Japan

During the study period, there were 10 cases of neonatal HSV infection. Therefore, the incidence of neonatal HSV infection in Japan was about 1 in 1.4 × 10^5^ live births.

### 3.2 Characteristics of neonates in 10 cases of HSV infection

Table 1 shows the maternal and neonatal characteristics of the 10 cases of neonatal HSV infection. The average maternal age was 27.6 ± 6 years, and 2 mothers were in their teens. One case delivered at 29 weeks because of premature rupture of the membranes, while the other 9 cases delivered at term.

**Table 1:** Clinical characteristics of mothers in 10 cases of neonatal herpes simplex virus infection in Japan.

Case	Maternal age (years old)	Past history of herpes simplex virus infection	Gestational weeks at delivery (weeks)	Premature rupture of the membranes	Maternal symptoms indicating viral infection around the time of delivery	Delivery mode
1	16	No	41	No	No	Vaginal delivery
2	21	No	29	No	No	Cesarean delivery
3	17	No	38	Yes	No	Vaginal delivery
4	31	No	37	No	No	Vaginal delivery
5	32	No	37	No	Genital ulcer	Cesarean delivery
6	28	No	38	No	No	Cesarean delivery
7	31	Yes (genital herpes simplex virus infection)	39	No	Bilateral nipple ulcers (postpartum)	Vaginal delivery
8	34	No	39	No	Fever ≥ 38℃	Cesarean delivery
9	34	No	39	No	Fever ≥ 38℃	Cesarean delivery
10	32	No	40	No	No	Vaginal delivery

In this study, 3 mothers had cesarean section because of the symptoms of viral infection such as genital ulcers or fever (≥ 38℃), while 2 had cesarean sections for other obstetric indications unrelated to the infection. One mother had ulcers on both nipples after vaginal delivery. She was the only one with a history of genital HSV infection.

In this study, the average timing of the onset of neonatal HSV infection was 4.1 ± 3 days after delivery. Four neonates had disseminated infections, 2 had central nervous system (CNS) infections, and 4 had skin/eye/mouth disease of HSV infection as shown in Table 2.

**Table 2:** Clinical characteristics of neonates in 10 cases of neonatal herpes simplex virus infection in Japan.

Case	Method of birth	Postpartum days of the onset of neonatal herpes simplex virus infection (days)	Disease type of neonatal herpes simplex virus infection	Herpes simplex virus type of neonatal infection
1	Vaginal birth	4	Disseminated infection	Type Ⅰ
2	Cesarean birth	4	CNS infection	Type Ⅰ
3	Vaginal birth	6	Disseminated infection	Type Ⅰ
4	Vaginal birth	0	Skin/eye/mouth disease	Unknown
5	Cesarean birth	0	Skin/eye/mouth disease	Unknown
6	Cesarean birth	10	CNS infection	Type Ⅰ
7	Vaginal birth	4	Skin/eye/mouth disease	Type Ⅰ
8	Cesarean birth	5	Disseminated infection	Type Ⅱ
9	Cesarean birth	5	Disseminated infection	Unknown
10	Vaginal birth	3	Skin/eye/mouth disease	Unknown

CNS, central nervous system.

## 4 Discussion

In this study, the incidence of neonatal HSV infection in Japan was about 1 in 1.4 × 10^5^ live births. In addition, we analyzed the characteristics of the mothers whose neonates had HSV infection to examine the risk of developing neonatal HSV infection. However, we could not find any other characteristic maternal findings common to cases of neonatal HSV infection except that the average age of mothers was slightly lower than that of Japanese average (27.6 vs. 30.7 years) (14).

Recently, neonatal HSV infection was reported to be identified at an incidence of 1 in 9 × 10^5^ live births in Denmark (15) and 1 in 0.4 × 10^5^ live births in Germany (16). There were no significant differences in neonatal HSV prevention measures, such as the use of prophylactic antiviral medications and prophylactic cesarean delivery in these countries (9, 15, 16). However, there may be room for improvement in strategies for the prevention of neonatal HSV infection in Japan.

To date, risk factors for neonatal HSV infection included vaginal delivery, first HSV infection, and prolonged premature rupture of the membranes (9, 17). One previous large-scale study in 2009 reported that HSV was isolated from the genital tract of the mother during delivery and that primary infection of HSV was a risk factor for neonatal HSV infection (18). In this study, it is considered that all cases would have been managed according to the Japanese guidelines (9), and since no case-control study was conducted, it is not possible to assess whether management according to the Japanese guidelines, including prophylactic cesarean section, is effective for preventing neonatal HSV infection. However, the current results, as well as those of other reports (1–4), reconfirmed that many mothers of neonates with HSV infection had no symptoms of the infection.

In this study, only 3 of the 10 mothers had symptoms of HSV at delivery, including one mother with a genital ulcer. Despite the fact that prophylactic cesarean delivery was performed for the 3 women as indicated by the guidelines (9), it did not prevent the development of neonatal HSV infection. Therefore, additional factors to prevent neonatal HSV infection in Japan should be examined in further studies.

In cases of neonatal HSV infection, the initiation of long-term antiviral suppressive therapy at an early stage of the disease has been reported to facilitate significant improvement in morbidity (3); however, the initial action might be delayed in cases without findings of HSV infection in the mother. Mothers of infants who acquire neonatal HSV infection sometimes lack histories of clinically evident genital HSV infection; therefore, clinicians should also recognize asymptomatic primary genital HSV infections (19). Based on the current results, the incidence of neonatal HSV infection might be low in Japan; however, we always have to keep in mind that neonatal HSV infection may develop under any circumstances. Therefore, at this point in time, it is important when mothers feel somewhat strange regarding their neonates and infants, and they should always communicate such feelings to medical professionals (13). In addition, medical professionals must always listen to the mother's complaints.

We understand the serious limitation of the short study period: the prevalence of HSV is low, and the 2-year study period may not have been sufficient to reflect the characteristics of neonatal HSV infection in Japan. Also, because reporting cases of HSV infections has not been mandatory in Japan, it may be challenging to increase awareness of the reporting of HSV infection. In addition, it was not possible to account for confounders concerning individual factors associated with neonatal HSV infection. There is also a possibility of response bias because the questionnaire was aimed at obstetric institutes. Although it may be important to identify the route of neonatal HSV infection, we could not identify trends in the risk of developing neonatal HSV infection in mothers.

A further study with the accumulation of many more cases of neonatal HSV infection may promote prevention in the future; however, in this study, only the need for awareness of the indeterminate complaints of neonates was recognized.

## 5 Conclusions

The incidence of neonatal HSV infection in Japan was about 1 in 1.4 × 105 live births. The current study could not address the merits of the Japanese guidelines on prophylactic cesarean section. In addition, we could not identify any characteristic findings common to cases of neonatal HSV infection in most mothers.

## Data Availability

The original contributions presented in the study are included in the article/Supplementary Material, further inquiries can be directed to the corresponding author.
